# Patient interpretation and implementation of air embolism prevention guidelines in hereditary hemorrhagic telangiectasia (HHT): a survey-based study

**DOI:** 10.1186/s42155-025-00620-4

**Published:** 2025-11-27

**Authors:** Kimberly Wei, Susan Shamimi-Noori, Theodore G. Drivas, Scott O. Trerotola

**Affiliations:** 1 Department of Radiology, Division of Interventional Radiology, Perelman School of Medicine, University of Pennsylvania, Philadelphia, PA USA; 2 Division of Translational Medicine and Human Genetics, Department of Medicine, Perelman School of Medicine, University of Pennsylvania, Philadelphia, PA USA

**Keywords:** HHT, PAVM, Bubble filter, International HHT Guidelines, Air embolism

## Abstract

**Background:**

To assess how patients interpret and adhere to the International HHT Guidelines’ recommendation to avoid intravenous (IV) air and to evaluate whether misinterpretation of this guidance as a strict requirement for in-line bubble filters may inadvertently hinder access to care. An anonymous 15-question survey was distributed to 7000 members of the HHT Research Network. The survey assessed awareness of the guideline, perceived necessity of bubble filter use, and the practical consequences of filter use. Responses were excluded if incomplete or submitted by individuals under 18 years old.

**Results:**

Of the 596 responses received (9% response rate), 446 met inclusion criteria. Most respondents (79%) were aware of the guideline, and 66% interpreted it as requiring use of an IV bubble filter. Notably, 16% of respondents reported refusing care, and 25% reported delaying treatment—most often patient-initiated—due to perceived filter requirements. The interventions affected included essential and, in some cases, urgent care. In total, 20 respondents (4%) reported experiencing a transient ischemic attack (TIA) during IV therapy; two of these occurred despite filter use, and none resulted in permanent deficits. Patients who did not use filters were significantly less likely to report difficulty accessing care (*p* < 0.05).

**Conclusions:**

Although the guideline advises caution in avoiding IV air, many patients interpret it as mandating bubble filter use. This misunderstanding has been linked to delays in necessary care, increased patient frustration, and limited treatment access. These findings underscore the importance of clearer communication and education around guideline intent to mitigate unintended consequences.

**Supplementary Information:**

The online version contains supplementary material available at 10.1186/s42155-025-00620-4.

## Background

Hereditary hemorrhagic telangiectasia (HHT) is a rare autosomal dominant vascular disorder characterized by telangiectasias and arteriovenous malformations (AVMs). It affects approximately 1 in 5000 individuals globally [1–4]. One of the more serious manifestations of HHT is pulmonary arteriovenous malformations (PAVMs), which occur in 15–50% of HHT patients [5–8]. PAVMs are particularly concerning due to the risk of paradoxical emboli, which may lead to systemic complications such as stroke or cerebral abscesses—events that occur in up to 36% of patients with PAVMs [1, 9].

The risk of air embolism during intravenous (IV) procedures has long been a concern in this population. Air embolism is most likely to occur during invasive procedures such as IV-line placement, surgery, or embolization therapy, and procedural precautions are universally recommended to minimize this risk. While the incidence of clinically apparent air embolism in HHT patients is not precisely quantified in the literature, it is a recognized and serious complication [10–12]. In our survey sample, the estimated true prevalence of PAVMs was approximately one-third (≈33%), which falls within the reported 15–50% range and supports the representativeness of the respondents to the broader HHT population. The literature consistently emphasizes paradoxical embolic events as a major source of morbidity in HHT patients with PAVMs.

In response to these concerns, the 2009 International HHT Guidelines recommended the “careful avoidance of intravenous air bubbles… to prevent cerebral air embolism,” and noted that “this could include an in-line filter… These precautions should be followed life-long, regardless of size of PAVMs, even once PAVMs are treated” [6]. However, the language of this recommendation was somewhat vague—phrases such as “could include” left room for interpretation, leading many providers and patient organizations to treat in-line filter use as mandatory rather than optional. This ambiguity, coupled with reinforcement from advocacy organizations such as CureHHT, which previously stated that “a 0.22 micron IV filter is recommended to keep air out of the IV line [13],” contributed to widespread adherence to this practice. Even after the 2020 guideline update, which omitted explicit mention of in-line filters [14], the perception among many patients and providers remained unchanged. In some cases, providers later began advising patients to stop using filters, but a survey of HHT Centers of Excellence revealed wide variation in clinician practices regarding filter use in HHT patients with PAVMs, with about half of physicians recommending filter use and half not—underscoring ongoing inconsistency in care implementation [15]. While preventing thromboembolic events remains a key goal, concern has grown that the perceived necessity of filters may lead to unintended consequences, including treatment delays, increased logistical burdens, and heightened patient anxiety.

A targeted PubMed search (“HHT and cerebral embolism and air bubble filter” and “HHT and cerebral embolism and PAVM”) yielded no studies directly evaluating the effectiveness of IV filters in preventing embolic events. Furthermore, there is little research on how patients interpret or respond to these guidelines. This study aims to fill that gap by assessing how the air bubble precaution guideline is understood and implemented by patients with HHT, and whether it contributes to disruptions in care. As a survey-based study, it is observational in nature and cannot establish causation, but it seeks to describe the real-world implications of current guideline interpretation.

## Materials and methods

This is an institutional review board (IRB)-exempt, prospective, single-cohort survey-based study designed to evaluate the clinical impact of patients’ perception and interpretation of the HHT Guidelines’ recommendations for individuals with PAVMs. The study aimed to assess (1) awareness of the guidelines, (2) interpretation and adherence, (3) feasibility and impact on care delivery, and (4) any associated adverse outcomes.

To address these objectives, data were collected via an online survey distributed over a two-week period in August 2023. The target population included individuals with HHT aged 18 years or older. A survey link was distributed to approximately 7000 patients enrolled in the Cure HHT Research Network’s registry, which includes individuals receiving care at HHT centers worldwide. To enhance participation, a single promotional post was also shared via Cure HHT social media channels during the collection period. No participation incentives were provided, as the anticipated time burden was minimal.

The survey (Supp. Figure 1) consisted of 15 questions developed to evaluate the domains aligned with the study objectives: awareness, adherence, feasibility, efficacy, and potential downsides of the air bubble filter recommendation. Key variables included self-reported awareness of the guideline, whether patients interpreted the recommendation as a mandate to use air bubble filters, any delays in treatment or imaging studies due to filter use, and adverse events such as transient ischemic attacks. Two Likert scale questions assessed perceived frequency of events or difficulties, and respondents were given the opportunity to provide optional free-text comments.

Survey data were collected and managed using Qualtrics, an online survey and distribution platform hosted by the home institution. Exclusion criteria included incomplete surveys (> 50% of questions unanswered) and respondents under the age of 18. The final response rate was 6%, introducing potential concerns about selection bias and representativeness. To assess the potential impact of nonresponse bias on prevalence estimates, a sensitivity analysis was performed. Given a 6% response rate, we recalculated the estimated overall PAVM prevalence under varying assumptions about the prevalence among nonrespondents.

Following data export, descriptive statistics were used to summarize responses. Univariable and bivariate analyses were performed using chi-square tests and validated through cross-tabulation to examine associations between awareness, adherence, and reported outcomes. Responses to Likert scale questions (Q4 and Q14) were dichotomized between “not frequently” and “frequently,” using a cutoff between “sometimes” and “about half the time.” Statistical significance was defined as *p* < 0.05. All analyses were conducted using SAS 9.4 (SAS Institute Inc., Cary, NC).

## Results

In total, 7000 patients in the HHT network were sent surveys, of which 596 responded (9%). Given that at most 50% of patients with HHT have PAVMs, this response rate is likely underestimated, and the actual rate is probably in the vicinity of 18%. With an observed respondent PAVM prevalence of 33%, the estimated overall prevalence varied from 16.1 to 49.0% depending on the assumed prevalence among nonrespondents. If nonrespondents had a low prevalence of 15%, the overall prevalence decreased to 16.1%; if they had the same prevalence as respondents (33%), the overall estimate remained 33.0%; and if nonrespondents had higher prevalence values (40–50%), the overall estimate increased to 39.6–49.0%. Across this plausible range, the estimated prevalence remained within previously reported values for HHT cohorts, supporting the representativeness of the respondent sample. It is also possible that selection bias influenced the findings, as respondents may have been more motivated to participate due to prior negative or confusing experiences with IV precautions or guideline interpretation. One hundred fifty responses were excluded according to the exclusion criteria, leaving a total of 446 responses (6%). Out of those responses, 354 (79%) patients were aware of the HHT guideline on air bubbles, while 80 (18%) were not (Q2). Ten (2%) patients responded as “Other” with a majority stating they are aware of the guideline but were confused on whether it is to be applied or has changed. Two (0.4%) patients left the response blank. Two hundred ninety-five (66%) patients stated they adhere to the perceived mandated air bubble filter recommendation while 137 (31%) patients do not adhere, and 14 (3%) patients left it blank (Q3). There are fewer people who adhere to the guideline than those who are aware. From the data, there were 67 patients (15%) who are aware but knowingly do not use air bubble filters.

**Fig. 1:**
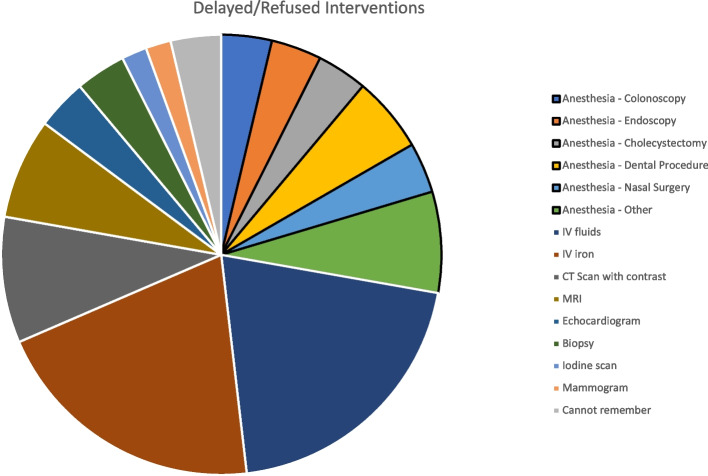
Refused/delayed medical interventions due to unavailability of bubble filter

Questions 4–10 asked about the availability of air bubble filters for HHT patients and its impact on receiving medical interventions. Fifty-four percent of patients were able to obtain a filter more than half the time as answered in Q4. Findings on filter impact on refusing or delaying treatments are summarized in Table 1. For patients who were initially refused an IV infusion, 23 patients agreed to proceed with IV infusion without a filter, while 14 had to either reschedule or be referred elsewhere for their infusion, and 7 patients had to find their own bubble filters for their treatments as answered in Q6. The refused/delayed interventions from question 8 included anesthesia for procedures such as endoscopies, dental procedures, and emergent surgeries as answered. Other interventions were IV infusions including iron infusions and imaging studies. The results are summarized in Fig. 1. Most of the treatment delays were due to the patient’s discretion (74) vs. the healthcare provider’s (46) as answered in Q10.

**Table 1:** Effect of filter availability on treatment access (Q5, 7, 9)

	**Yes,** *n* (%)	**No,** *n* (%)	**Blank answer,** *n* (%)
Have you been refused an IV infusion because of no available filters?	72 (16%)	363 (81%)	11 (3%)
Have you ever refused a needed study/treatment because of no available filters?	72 (16%)	363 (81%)	11 (3%)
Have you ever had a treatment rescheduled/delayed because of no available filter?	113 (25%)	309 (70%)	24 (5%)

Questions 11–13 involved evaluating occurrence of adverse events related to the bubble filter use. Patients were specifically asked about the occurrence of stroke or transient ischemic attack (TIA) while receiving IV therapy. Twenty (4%) patients reported to have had a TIA while receiving IV therapy. Out of those 20, two patients had an air bubble filters in place during this event while 11 did not. Seven responded with “Other,” with the majority unable to remember. Out of the 20 who did experience a TIA, none of them had any permanent effect from the incident.

Question 14 evaluated how bubble filter use affected the degree of difficulty in accessing treatment. Thirty-eight percent of patients reported that using air bubble filters made accessing treatment more difficult greater than half the time, while 25% of patients reported that following the guidelines never made it more difficult to access treatment. However, when stratifying results by patients who use vs. do not use bubble filters, there were significantly more people who do use filters who never found it difficult to access treatment (*p* < 0.001). The results summarized in Table 2.

**Table 2:** Frequency of filter use making accessing treatment more difficult (Q14)

	Patients who **want to use IV filter**, *n* (%)	Patients who **do not use IV filters,** *n* (%)	***P*-value**
Total	*N* (66%)	*N* (31%)	
Always	30 (10%)	17 (12%)	0.3
Most of the time	52 (18%)	15 (11%)	0.2
Half of the time	31 (11%)	5 (4%)	0.04*
Sometimes	95 (32%)	9 (7%)	< 0.001*
Never	75 (25%)	71 (52%)	< 0.001*
Blank	12 (4%)	20 (15%)	

Patients were also given one free-text section (question 15) at the end of the survey to express any thoughts on the guidelines. Forty-six responses reported frustration when trying to adhere to the perceived recommendation due to lack of filter availability or healthcare provider awareness. Many patients were also confused about the guidelines and expressed distress about the uncertainty. Representative comments can be seen in Table 3.

**Table 3:** Representative comments from patients

Frustration with acquiring/using filters	Confusion about the recommendation
The day of my surgery I reminded the small outpatient hospital I would need one. They had to send someone to a larger hospital to get one and charged me a “mediflight” cost	They believe because mine are micro they don’t have to worry about the filter
When I request a filter prior to an IV, no one knows what I am talking about. I have only been able to get one and that was asked for ahead of time for a planned outpatient surgery. For any other procedure (ex. would be a colon scope), I have had to go ahead without having a filter because they do not have them	The air bubble filter information is not always clear. The docs and nurses do not know what we are talking about when we ask them. And it is difficult to remember exactly what we need…
It’s always a battle	I used air filters for about 20 years until my current Dr advised it isn’t necessary
I’ve had at least 10 surgeries and I have to tell them every time I need it and usually have to wait until they get one. It’s very frustrating…	A lot of places seem confused and I have to convince them it is necessary

Bivariate analyses showed there was no significant difference between awareness (Q2), adherence of perceived recommendation (Q3), or filter use (Q4) and stroke prevalence (Q11) (*p* = 0.72, *p* = 0.25, and *p* = 0.56, respectively). There was a significant difference between filter use (Q3) and refusing (Q7) or delaying (Q9) an intervention (*p* =  < 0.0001). There also was a significant difference between difficulty accessing treatment (Q14) vs refusing or delaying treatment (*p* =  < 0.0001).

## Discussion

This survey-based study explored patient interpretations of the International HHT Guidelines’ air bubble recommendation, particularly regarding the use of IV air bubble filters. The results demonstrate widespread adherence to what many patients perceive as a strong recommendation for filter use. This perception has been associated with unintended consequences, including delays in necessary treatment, refusal of care, and increased patient distress—without clear evidence of clinical benefit.

While the current guidelines do not explicitly mandate IV bubble filters, historical context has contributed to patient confusion. The 2009 guidelines recommended “careful avoidance of intravenous air bubbles… [which] could include an in-line filter,” language that was widely interpreted as a directive for filter use. Although the 2020 update softened this to “take extra care to avoid IV air,” this change was not clearly highlighted, and the older, more prescriptive language continued to shape patient and provider behaviors [6, 14]. The Cure HHT website previously reinforced this interpretation by explicitly recommending a 0.22 micron IV filter, further cementing the perceived necessity of filters [13].

Our findings suggest that this longstanding confusion has had meaningful clinical implications. Patients who use filters were statistically more likely to have refused, delayed, or rescheduled needed treatments due to filter unavailability. Alarmingly, the treatments impacted included interventions essential to standard care, such as IV iron therapy, contrast-enhanced imaging, and even urgent surgical procedures. Although patients without PAVMs were not excluded, the responses likely represent a cohort of HHT patients with PAVMs, based on their awareness and engagement with the air bubble recommendation.

The potential benefits of filter use in HHT patients with PAVMs must be weighed against its limitations and trade-offs. Although paradoxical embolism is a known risk, prior imaging studies have reported cortical infarctions in about 14% of patients with a single PAVM and 12.5% of HHT patients overall [16, 17]. In contrast, our survey found a much lower incidence of self-reported air embolism events (4%). This discrepancy likely reflects differences in detection methods—imaging can identify both symptomatic and silent infarcts, whereas patient reports capture primarily clinically apparent events. Silent brain infarcts have been documented in nearly 10% of HHT patients without prior stroke or TIA, suggesting a notable burden of asymptomatic cerebral injury that may go unrecognized. The low rate of self-reported TIA/stroke in our survey could therefore reflect under-recognition rather than true absence of events, highlighting the importance of imaging and clinical monitoring to fully characterize embolic risk [18].

Importantly, our data showed no significant difference in embolic events between patients who used filters and those who did not, suggesting that filter use may not substantially reduce the risk of symptomatic TIAs or strokes. Some patients with filters still reported TIAs, though these were unconfirmed clinically, and the lack of lasting neurological deficits offers some reassurance. Given the self-reported, cross-sectional nature of the data and potential biases, causal conclusions cannot be drawn. Nonetheless, these findings raise questions about the effectiveness of filters in preventing embolic events in this population. Considering the multifactorial nature of embolic risk in HHT, including PAVM characteristics and patient-specific factors, filter use should be considered within the broader clinical context. Further prospective research combining clinical and imaging data is needed to better understand the role of filters in managing these patients.

Beyond clinical outcomes, patients highlighted the logistical and emotional burden of filter use. Those who reported frequent difficulty accessing treatment were disproportionately users of filters, while most of the patients who reported never having difficulty accessing treatment do not use filters. Anecdotal comments revealed that some patients had to advocate for filter use in unfamiliar clinical settings, leading to negative healthcare experiences. For rural hospitals or smaller clinics, where filters may not be readily available, both patients and providers faced additional time, effort, and cost burdens to comply with the perceived recommendation. These findings underscore the need for provider education initiatives, particularly targeting community and rural settings, to ensure safe, evidence-informed IV care without unnecessary barriers.

Shared decision-making emerges as a practical solution moving forward. Engaging patients and providers in discussions about actual embolic risk, benefits and limitations of filters, and logistical considerations can help tailor care to individual circumstances, reduce anxiety, and minimize treatment delays. Combining guideline recommendations with clear communication and patient-centered decision-making may improve adherence while mitigating unintended consequences.

As with any survey-based investigation, this study has inherent limitations. Sampling bias, non-responder bias, and response bias may have influenced the findings. Self-selection bias is also a concern, as patients who were more engaged, motivated, or who had strong feelings about the recommendation may have been more likely to participate. Because responses were anonymous, we could not ensure even representation across different HHT centers or confirm that the sample accurately reflected the geographic and clinical diversity of the broader HHT population. We were also unable to analyze factors such as age, sex, geographic location, clinical severity, or healthcare system characteristics (e.g., urban vs. rural, private vs. public) that could influence both patient experiences and interpretation of the air bubble guideline. Additionally, the survey instrument was not formally pre-tested or validated prior to administration. Consequently, we cannot confirm that all questions were interpreted consistently across participants or that the survey fully captured the constructs of interest. These limitations may contribute to measurement error and should be considered when interpreting the findings, particularly regarding self-reported experiences. The overall response rate was relatively low, though this is not uncommon in survey-based research [19]. Furthermore, responses were dependent on memory and may be subject to recall error. The accuracy of self-reported experiences, such as embolic events, cannot be independently verified. Nevertheless, the final sample size achieved statistical significance for a population of 7000, using a 95% confidence level and 5% margin of error, allowing cautious generalization of these findings to the population of HHT patients with PAVMs [20].

Although the International HHT Guidelines do not explicitly require IV bubble filter use, this study highlights widespread misinterpretation among patients, leading to avoidable delays in care and increased emotional distress. Given the limited data on clinical benefit and the real-world consequences of this misunderstanding, clearer communication from guideline authors and HHT advocacy groups is needed. Educational efforts for both patients and providers should emphasize safe air-free IV techniques that do not depend on specialized filters. Future research should further investigate the actual risk reduction offered by filters and evaluate strategies to reduce unnecessary barriers to care.

## Supplementary Information

Additional file 1. Survey questions and answer choices.

## Data Availability

The data that support the findings of this study are available from the corresponding author, SOT, upon reasonable request.
