# Supplementary material for: Patient interpretation and implementation of air embolism prevention guidelines in hereditary hemorrhagic telangiectasia (HHT): a survey-based study
Source: CVIR Endovasc. 2025 Nov 27;8:104. doi: 10.1186/s42155-025-00620-4 (PMC12657698; doi:10.1186/s42155-025-00620-4)
Supplement: Supplementary file 1 — Additional file 1. Survey questions and answer choices. [file 42155_2025_620_MOESM1_ESM.docx]

Unanticipated Consequences of Bubble Filter Recommendation for Patients with PAVM in HHT Guidelines

Start of Block: Default Question Block

Q1 Are you over the age of 18?

- Yes (1)
- No (2)

Skip To: End of Survey If Are you over the age of 18? = No

Q2 Are you aware of the HHT guidelines recommendation for an air bubble filter when intravenous access (an IV) is in place?

- Yes (1)
- No (2)
- Other (3) __________________________________________________

Q3 Do you adhere to these guidelines?

- Yes (1)
- No (2)

Q4 How often are you able to obtain an air bubble filter when you need an IV?

- Always (1)
- Most of the time (2)
- About half the time (3)
- Sometimes (4)
- Never (5)

Q5 Have you ever been refused an IV infusion because a bubble filter was not available?

- Yes (1)
- No (2)

Skip To: Q6 If Have you ever been refused an IV infusion because a bubble filter was not available? = Yes

Skip To: Q7 If Have you ever been refused an IV infusion because a bubble filter was not available? = No

Q6 If yes, what was the alternative management strategy?

________________________________________________________________

Q7 Have you ever refused to get a needed study or treatment because a bubble filter was not available?

- Yes (1)
- No (2)

Skip To: Q8 If Have you ever refused to get a needed study or treatment because a bubble filter was not available? = Yes

Skip To: Q9 If Have you ever refused to get a needed study or treatment because a bubble filter was not available? = No

Q8 If so, what study or treatment?

________________________________________________________________

________________________________________________________________

________________________________________________________________

________________________________________________________________

________________________________________________________________

Q9 Have you ever had a study or treatment delayed/rescheduled because a bubble filter was not available?

- Yes (1)
- No (2)

Skip To: Q10 If Have you ever had a study or treatment delayed/rescheduled because a bubble filter was not availa... = Yes

Skip To: Q11 If Have you ever had a study or treatment delayed/rescheduled because a bubble filter was not availa... = No

Q10 If so, was this decision yours or the health professional's?

- Patient's (1)
- Health Professional's (2)

Q11 Have you ever had a stroke/TIA (mini stroke) while receiving IV therapy?

- Yes (1)
- No (2)

Skip To: Q12 If Have you ever had a stroke/TIA (mini stroke) while receiving IV therapy? = Yes

Skip To: Q14 If Have you ever had a stroke/TIA (mini stroke) while receiving IV therapy? = No

Q12 If so, was there a bubble filter in place?

- Yes (1)
- No (2)
- Other (please specify) (3) __________________________________________________

Q13 If yes, did you have a permanent effect from that event?

________________________________________________________________

________________________________________________________________

________________________________________________________________

________________________________________________________________

________________________________________________________________

Q14 How often has following the bubble filter recommendation made accessing treatment more difficult?

- Always (1)
- Most of the time (2)
- About half the time (3)
- Sometimes (4)
- Never (5)

Q18 If you have any additional comments, please include them here.

________________________________________________________________

________________________________________________________________

________________________________________________________________

________________________________________________________________

________________________________________________________________

End of Block: Default Question Block

**Online Resource.** Survey Questions and Answer Choices.

**Title:** Patient Interpretation and Implementation of Air Embolism Prevention Guidelines in Hereditary Hemorrhagic Telangiectasia (HHT): A Survey-Based Study

**Journal Name:** Cardiovascular and Interventional Radiology

**Author names:** Kimberly Wei, BS, Susan Shamim-Noori, MD, MSEd, Theodore G. Drivas, MD, PhD, and Scott O. Trerotola, MD

**Corresponding Author:**

Scott O. Trerotola, MD

Department of Radiology, Division of Interventional Radiology; Perelman School of Medicine, University of Pennsylvania, Philadelphia, PA, USA

Email: streroto@pennmedicine.upenn.edu
